# Comparing BPS and BPA: Cardiovascular Effects in Female Rat Hearts

**DOI:** 10.1289/ehp.123-A157

**Published:** 2015-06-01

**Authors:** Nate Seltenrich

**Affiliations:** Nate Seltenrich covers science and the environment from Petaluma, CA. His work has appeared in *High Country News*, *Sierra*, *Yale Environment 360*, *Earth Island Journal*, and other regional and national publications

Bisphenol A (BPA) is being removed from many consumer products because of concerns about the chemical’s potential to disrupt the endocrine system.^1^ A chemical analog known as bisphenol S (BPS) has been adopted as an alternative in products such as water and baby bottles, thermal paper, and linings of metal cans.^2^ In this issue of *EHP,* a new study shows that BPS has nearly identical impacts on the rat cardiovascular system as those previously reported for BPA by members of the same team.^3^

“When we started this project, we didn’t know what to expect because there was no data available on the cardiovascular effect of BPS,” says lead author and recent University of Cincinnati graduate Xiaoqian Gao. “We didn’t know whether it would have no effect, or a stronger effect, or a weaker effect.”

**Figure:**
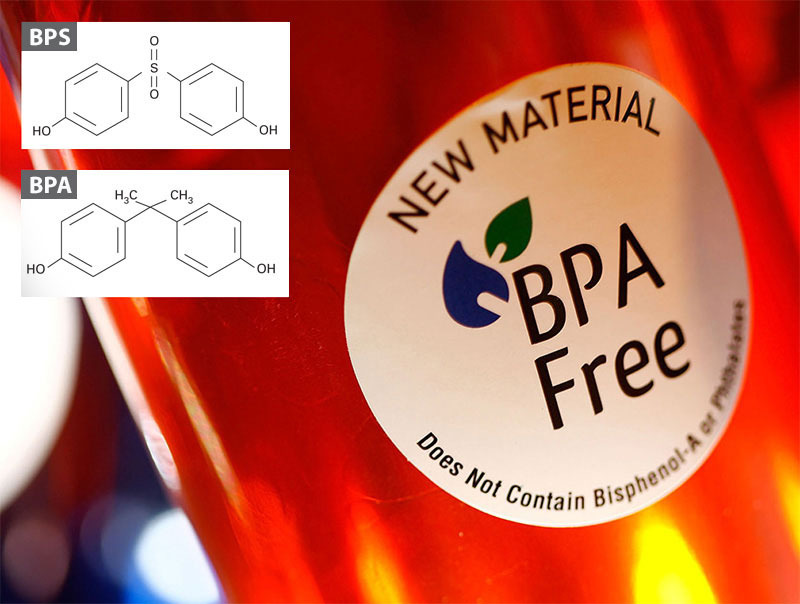
Consumer demand and research concerns prompted the removal of BPA from many consumer products. But one of its replacement, BPS, is proving to behave quite similarly in cells and organisms. © David McNew/Getty Images

The researchers performed back-to-back studies to evaluate the short-term effects of BPA and BPS on adult rat whole-heart preparations and ventricular myocytes. The first study, on BPA, was published in 2011.^4^ The current study applied the same model to BPS.

“We show, using cardiac end points, that BPS has remarkably similar effects on the heart and on cardiac cells as what we previously described for BPA,” says senior author Hong-Sheng Wang, Gao’s faculty adviser. “And the two have almost identical potencies.”

Both studies also found that effects occurred only in female tissues, with no detectable effects in male hearts or ventricular myocytes. This is consistent with the varying effects of estrogenic chemicals on male and female mammals, Hong-Sheng says.

For both studies, the researchers used ventricular myocytes to study the impact of BPA and BPS on the development of aberrant spontaneous excitations, or “triggered activities” (one of the chief mechanisms that can give rise to arrhythmic activity), as well as on calcium handling (an indicator of cardiac physiology). They used whole-heart preparations to study impacts on electrical rhythm. For the current study, samples were treated with BPS alone, the stimulant heart drug isoproterenol, BPS plus isoproterenol, or BPA plus isoproterenol.^3^

In female whole hearts, a dose of 10^–9^ M BPS increased the frequency of premature ventricular beats—the most common form of arrhythmia in humans^5^—to 8.83 events per 20 minutes, versus 0.87 events with isoproterenol alone. BPS alone did not trigger arrhythmia events but did result in moderate heart rate increases. Likewise, triggered activities were recorded in 30% of ventricular myocytes exposed to 10^–9^ M BPS, versus 6.6% of controls; doses of 10^–12^ M and 10^–6^ M did not increase the percentage, creating an inverted U dose–response curve.^3^

In female ventricular myocytes, doses of 10^–9^ M and 10^–8^ M BPS enhanced fractional shortening—a measure of ventricular function—to the same degree as 10^–9^ M BPA. And the researchers found BPS and BPA had similar effects in quickly and temporarily increasing the phosphorylation of calcium-handling proteins, including ryanodine receptor and phospholamban.^3^ Much more research is needed to determine how these responses may translate to effects in living organisms.

As with BPA before it, human exposure to BPS is becoming widespread. A 2012 study detected BPS in the urine of 81% of 315 people sampled, including males and females aged 2–84 years living in the United States and seven Asian countries.^6^

Johanna Rochester, a research associate with The Endocrine Disruptor Exchange and lead author of a forthcoming *EHP* review of health effects of BPS and another analog called bisphenol F,^7^ says that while the Gao study was not included in her paper as it had not yet been published, it arrives at a similar conclusion—that experimental effects of BPS are similar to those of BPA.

The new study is also the first to evaluate the cardiovascular effects of BPS and compare them with those of BPA, Rochester says. “When people think of BPA and endocrine disruption in general, they tend to think of reproduction and thyroid function,” she says. “But this is showing that endocrine disruptors … can disrupt many different systems.”
